# Fuzzy based priority aware task scheduling optimization for mobile edge computing environments

**DOI:** 10.1038/s41598-025-23690-9

**Published:** 2025-11-14

**Authors:** Pei Lin

**Affiliations:** College of Digital Media, Lanzhou University of Arts and Science, Lanzhou, 730010 Gansu China

**Keywords:** Mobile edge computing, Scheduling, Greedy algorithms, Fuzzy logic, Energy consumption, Latency, Energy science and technology, Engineering, Mathematics and computing

## Abstract

Mobile Edge Computing (MEC) is an innovative solution designed to address key challenges in mobile cloud computing, including latency, limited capacity, and resource constraints. The primary objective of MEC is to enable dynamic scheduling and efficient resource allocation with minimal cost. This paper proposes a three-tier system architecture comprising mobile devices, edge computing nodes, and traditional cloud infrastructure. It introduces two methods for task offloading and scheduling. For task allocation on mobile devices, the system leverages the **G**reedy** A**uto-**S**caling **O**ffloading algorithm, which prioritizes high-energy-consuming tasks to enhance energy efficiency. On the edge computing layer, a dynamic scheduling approach based on fuzzy logic is presented, which ranks and allocates tasks according to two specific criteria. Numerical evaluations demonstrate that, compared to existing alternatives, the proposed method significantly reduces task waiting time, latency, and overall system load, while maintaining system balance with minimal resource consumption. Moreover, the proposed system achieves up to ~ 64% reduction in battery consumption in our simulated environment compared with local execution. The results also indicate that over 93% of tasks are successfully executed within the edge environment.

## Introduction

Currently, mobile phones have a pivotal position in individuals’ lives. The advancement of mobile phone networks and the Internet of Things (IoT) has presented numerous issues in computing, storage, and network management^1–3^. These difficulties encompass significant delays, restricted storage capacity, resource-constrained devices, the necessity for uninterrupted intermittent service, and heightened security, which are not fully addressed by cloud-based architecture^4,5^. Mobile devices, including smartphones, tablets, and wearables, are active components of the IoT^6^, wherein numerous sensors are interconnected with other devices in residential, commercial, and transportation environments using contemporary technological networks^7,8^. The Fifth Generation (5G) of mobile phone systems networks was developed to expedite the advancement of apps for mobile devices^9^.

5G networks prioritize the implementation of advanced technologies, including mobile cloud computing (MCC) and MEC, to enhance flexibility and enable real-time processing for applications on mobile devices^10^. These applications, such as augmented reality, gaming, and facial recognition, necessitate substantial computational power and consequently demand significant energy during processing.

The allocation of resources in cloud computing, resulting from distant user access^11^, induces latency in response times; thus, there is a demand for innovative technologies to enhance speed and efficiency in delay-sensitive and real-time applications. Since 2016, the European Telecommunications Standards Institute (ETSI) has developed a reference framework and architecture for Multi-Access Edge Computing. This architecture facilitates services including the execution of dynamic programs and the real-time dissemination of radio network information^12,13^.

Load balancing for resource optimization in edge environments^14^, cloudlet-based approaches for mobile augmentation and resource optimization^15^, and a cloudlet federation-based framework have been reviewed, which aims to improve resource allocation and increase efficiency in edge networks^16^. The objective is to link millions of real-time and latency-sensitive applications at the network’s edge, thereby reducing energy consumption in mobile devices, accelerating the execution of real-time tasks, and expanding data storage and application processing capacity. This platform presents both advantages and challenges^17^.

Selecting appropriate activities for transmission to MEC necessitates precision, as dispatching task sets over the network can incur elevated costs regarding battery energy consumption or network bandwidth for the mobile device^18^. A significant challenge in the MEC domain is the entry of tasks into this layer without prior knowledge^19^, or at least the process of obtaining adequate information regarding the criteria for prioritizing tasks is time-consuming, which is crucial for effective task scheduling^20,21^.

The dynamic prioritizing of jobs, with corresponding costs determined by the existing knowledge within the network, is a significant concern in task scheduling. This article discusses the edge layer connecting the cloud and mobile application programs, utilizing many virtual machines with several CPUs on the MEC side. Tasks may be performed locally on the mobile device or transmitted to MEC for execution. Tasks possess criteria; in this context, we underscore two specific criteria: energy usage and execution time.

This article emphasizes that understanding these two criteria is crucial for selecting and scheduling assignments. The optimal selection of tasks on the mobile phone to be transmitted to the MEC is accomplished by the GASO, which seeks to minimize energy usage on the device. Task scheduling on the MEC side is executed using the fuzzy technique.

This algorithm utilizes energy consumption and execution time as inputs for the fuzzy technique, with the task execution priority as the output. Fuzzy-based task prioritization (FPTS) use fuzzy logic to convert input data into inference variables. Fuzzy logic is an appropriate approach for work prioritization in contrast to traditional methods like first-in-first-out (FIFO), round robin (RR), and shortest job first (SJF).

We evaluate our suggested technique against this method across various characteristics, including waiting time, service level, and delay %. We implement a resource allocation policy at the edge that is contingent upon the active resources present in the network. In the absence of sufficient resources for a task within the network, the task is dispatched to the cloud for execution. The primary objective of this article is to transmit an appropriate collection of tasks from the device to the edge to minimize energy consumption in the device. Our objective at MEC is to implement dynamic work scheduling by utilizing minimal resources to decrease expenses. If a task is not performed in the MEC layer, it is forwarded to the upper layer, namely the cloud, for execution. The primary objectives of this paper can be encapsulated as follows:

- The fundamental objective of this study is to present GASO as an optimal loading method that enhances energy efficiency for each activity and accelerates performance on mobile devices by effectively distributing tasks to improve resource usage.
- Providing FPTS as a dynamic loading method based on the dual criteria of energy consumption and execution time, while implementing an optimal resource allocation policy with the minimal number of processors to prioritize tasks and enhance task execution speed on the edge, constitutes a significant aspect of scheduling in this study.
- A numerical analysis of evaluation results for parameters such as waiting time, probability of delay, service level, and system overhead, utilizing the minimum number of processors, compares the proposed scheduling method with exit methods based on order of arrival, round-robin, and shortest job first, assessing the extent of performance superiority of the method. A recommendation is provided over alternative methods.

This work improves MEC scheduling by combining a fuzzy priority scheduler (FPTS) with a GASO-based greedy discharge selection, with simultaneous energy-delay optimization under real-time constraints. In contrast to heavy DRL or iterative metaheuristics, our design (i) achieves polynomial time end-to-end $\text{O}\left(\text{n log n}\right)+\text{O}\left(\text{nm}\right)$ suitable for online decisions, (ii) employs a feasibility-first resource policy to ensure a valid allocation each slot, and (iii) directly encodes dual criteria into fuzzy rules for stable prioritization under workload volatility.

The subsequent sections of this article are as follows: In Part 2, we examine the research background, followed by a detailed description of the system model and relationships in Part 3. Sect. “Proposed method” presents the proposed strategy for work loading and scheduling, while Sect. “Evaluation” provides the simulation results. Ultimately, Sect. “conclusion” presents the summary and conclusion of the paper.

## Related Works

Due to IoT device resource limits, latency-sensitive Internet of Things (IoT) applications are rapidly expanding, shifting computing tasks from IoT users to MEC. IoT users must compete for MEC computing resources, especially for dependent tasks with short deadlines. However, most dependent task delegation techniques may not account for IoT user resource contention, which may limit system performance in multi-user environments.

In^22^, an auction-based dependent job assignment method improves work assignment for numerous IoT users. First, dependent task assignment is described as an NP-hard valuation maximization issue in computing resource trade that meets customers’ latency criteria. Next, the task graph structure and MEC state are combined to offer an honest auction mechanism termed greedy winner selection technique, which assigns winners a heuristic dependent task to boost task offloading efficiency. ^23^ presents a unique IoT device attack detection method employing ensemble-optimized deep transfer learning and the Whale Optimization Algorithm (WOA). Multiple ensemble-optimized deep transfer learning (DTL) models transfer knowledge from other areas to IoT data.

A cooperative vehicle-edge-cloud offloading approach utilizing V2V communication is presented in^24^. The approach makes use of a variety of resources, such as the cloud, edge, idle resource vehicles (ICRVs), and task requesting vehicles (TRVs). Multi-objective models and an enhanced NSGA-II algorithm are used to increase the stability and quality of task allocation. However, this study lacks a method for adaptive scheduling of priority tasks or the use of fuzzy logic, which is taken into consideration in the current work, and instead places a strong emphasis on network-level modeling and evolutionary optimization.

Numerous studies have been carried out in the areas of efficient task scheduling and MEC. For instance, in^25^, a method for pre-deployment of edge services based on user position prediction is introduced, resulting in lower latency and better quality of service. However, the task scheduling and adaptive prioritization concerns examined in this research are not covered in this work, which concentrates on the location-based component of services.

Another study in^26^ shows encouraging results in lowering energy consumption and focuses on the deployment of UAVs and edge connection for energy-efficient federated learning. However, this research is more focused on optimizing UAV–MEC communications and does not include a mechanism for scheduling prioritized activities or applying fuzzy logic.

An technique for fuzzy and uncertain data clustering with a strong capacity to handle uncertain data was suggested in the domain of fuzzy logic^27^. This approach is useful, but it can only be used for data clustering; it cannot be used directly for job scheduling in the MEC context.

The problem of allocating computational resources and offloading dependent activities in MEC is examined in^28^. In order to minimize latency and energy consumption, they provide a Deep Q-Network-based algorithm (JTOCRA-DQN) to solve an NP-hard problem by taking into account the maximum allowable task delay. Although DQN has been applied in a novel way, this work is restricted to resource optimization and dependent task modeling; it does not address task prioritizing, battery consumption control, or fuzzy techniques for dynamic scheduling. Additionally, a deep reinforcement learning (DQN) framework for scheduling production workshops with AGVs was presented in^29^, which proved successful in increasing productivity and decreasing machine idle time. However, the issues of energy consumption and MEC latency are not addressed by this approach, which is more suited for industrial applications.

To improve the task completion rate in MEC, a mobile-aware method to task scheduling is presented in^30^ that creates a task migration model (MINLP) and mimics the movement of mobile devices. They combined a genetic algorithm with rescheduling operator (PSOGAR) and particle swarm optimization to overcome this issue. However, because it relies on metaheuristic algorithms, this method has a significant computational cost in large-scale settings. Additionally, it lacks a way to manage work priority and lower mobile device energy consumption.

In a similar vein ^31^, offered a fuzzy multi-objective optimization model that balances efficiency and safety factors for scheduling flights of various aircraft types. This study’s drawback is that, despite its use of fuzzy logic, it ignores the resource-constrained and distributed contexts of MEC^32^. proposes a blockchain-based offloading system for augmented reality in UAV-MEC that balances rendering delay and energy usage. Fuzzy prioritization and adaptive scheduling methods are absent from this method, though. Lastly, the study^33^ presented a productive scheduling technique for SMT assembly factories that makes use of heterogeneous graphs and reinforcement learning. Despite its effectiveness, this study only looked at industrial businesses and ignored the unique problems with edge computing and mobile consumers.

An event-triggered fuzzy adaptive control technique is presented in^34^ to enhance the performance of cascaded PDE–ODE systems with actuator failures. An event-triggered dissipative tracking control method for network control systems with distributed delays was introduced in study^35^. The system’s stability under delay conditions was guaranteed by this method. A secure fuzzy T-S control technique under DoS attacks is introduced in^36^, strengthening the semi-automatic active suspension system’s defenses against dangers. This study illustrates how fuzzy logic can be used in unsafe settings. In a paper^37^, memory-based event-triggered adaptive control for fuzzy wind turbine systems was introduced in order to optimize performance under changing environmental conditions.

The suggested model aims to enhance task offloading in dynamic situations. A comparison of scheduling methods in mobile computing for existing tasks and the proposed approach is reviewed in Table 1.

**Table 1:** Comparison of scheduling methods in mobile computing for existing works and the proposed approach.

Ref	Models	Features	Environment
^22^	Auction-based dependent task assignment	job allocation mechanism for IoT–MEC	IoT–MEC
^23^	DTL with WOA	Develops an IoT device attack detection framework	IoT–edge security
^24^	V2V-edge-cloud offloading (NSGA-II)	Multi-objective optimization; no fuzzy/adaptive scheduling	Vehicle–edge–cloud
^25^	ESPD-LP (Location Prediction)	Reduces latency; ignores priority scheduling	MEC
^26^	UAV Deployment & Edge Association	Energy-efficient UAV–MEC; lacks fuzzy prioritization	UAV–MEC
^27^	Belief-based fuzzy clustering	Manages uncertain data; not for task scheduling	Fuzzy systems
^28^	JTOCRA-DQN	Minimizes latency/energy; no task prioritization	MEC
^29^	RL-based AGV scheduling	Improves productivity; not MEC-specific	Industrial scheduling
^30^	PSOGAR (GA + PSO)	Mobile-aware migration; costly, no priority/energy mgmt	MEC
^31^	Fuzzy multi-objective scheduling	Balances safety/efficiency; ignores MEC context	Transport scheduling
^32^	Blockchain-based AR Offloading	Balances delay/energy; lacks fuzzy/adaptive scheduling	UAV–MEC
^33^	Graph + RL scheduling	Efficient in SMT; not MEC-specific	Industrial scheduling
^34^	Event-triggered fuzzy adaptive	Improves PDE–ODE systems; not task scheduling	Control systems
^35^	Event-triggered dissipative tracking	Ensures stability with delays; not MEC scheduling	Control systems
^36^	Secure fuzzy T–S control	Robust under DoS; no MEC task focus	Control systems
^37^	Memory-based fuzzy control	Optimizes turbines; domain-specific	Control systems
Proposed	FPTS & GASO	Fuzzy priority scheduling + Greedy Auto-Scaling Offloading	MEC (three-tier)

## System model

The cloud, edge virtual machines, and mobile devices are all part of the standardized three-layer MEC architecture. The formula for time is $\text{t}=\text{1,2},\dots$ Each time interval involves a collection of tasks $\text{T}\left(\text{t}\right)=\left\{1,\dots ,\text{n}\left(\text{t}\right)\right\}$ with properties $\left({\text{d}}_{\text{i}}, {\text{s}}_{\text{i}}, {\text{e}}_{\text{i}}\right)$ as input: device-side energy factor ${\text{e}}_{\text{i}}$, compute cycles ${\text{s}}_{\text{i}}$, and input size ${\text{d}}_{\text{i}}$. Memory ${\text{r}}_{\text{k}}$, storage ${\text{u}}_{\text{k}}$, and CPU rate ${\text{c}}_{\text{k}}$ are all present in each edge virtual machine $\text{k}\in \left\{1,\dots ,\text{m}\right\}. {\text{K}}_{\text{i}\left(\text{t}\right)}\in \left\{\text{0,1}\right\}$ selects offload (1) over local (0) in a binary decision. An approved virtual machine that meets the requirements $\frac{{\text{c}}_{\text{k}}}{{\text{s}}_{\text{i}}}\ge 1$ and $\frac{{\text{r}}_{\text{k}}}{{\text{ram}}_{\text{i}}}\ge 1$ is given the task if offloading is performed. It is sent to the cloud otherwise. Reducing delay and energy consumption while preserving system equilibrium is the aim.

**Fig. 1:**
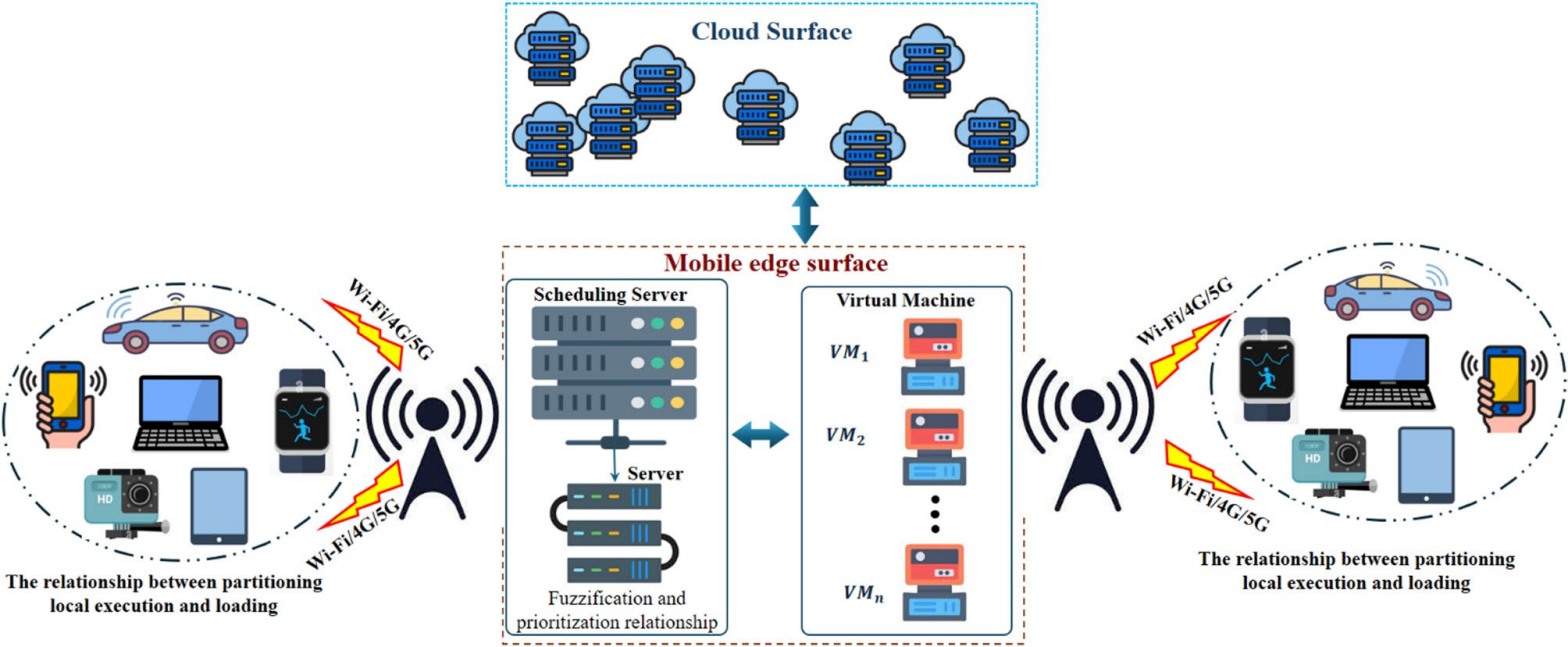
Proposed system model.

Our system comprises three tiers. At the most fundamental level, multiple entities are accountable for the GASO of the mobile device, and the interface determines the optimal set of tasks to dispatch to the edge. At the decision-making and task prioritization level for the second FPTS, the interface is established, and resource allocation is conducted at this level. If there are no active resources to allocate at this level, the tasks are sent to the cloud at the highest level. The model image of the proposed system is shown in Fig. 1.

### Assumptions

To make system modeling easier, this study makes a number of assumptions. In order to determine the transmission time of task $$ii$$, we first assume that the wireless link is stable throughout each slot. This means that $$T_{tr,i} \left( t \right) = {\raise0.7ex\hbox{${d_{i} }$} \!\mathord{\left/ {\vphantom {{d_{i} } {C_{vm} \left( t \right)}}}\right.\kern-0pt} \!\lower0.7ex\hbox{${C_{vm} \left( t \right)}$}}$$, where $$C_{vm} \left( t \right)$$ is the virtual machine layer’s transmission capacity at time t and $$d_{i}$$ is the input data size. Second, $$T_{local,i} \left( t \right) = {\raise0.7ex\hbox{${s_{i} }$} \!\mathord{\left/ {\vphantom {{s_{i} } {C_{local} \left( t \right)}}}\right.\kern-0pt} \!\lower0.7ex\hbox{${C_{local} \left( t \right)}$}}$$ is the expression for the local execution time. Where $$C_{local} \left( t \right)$$ is the available local processing capacity and $$s_{i}$$ is the number of computing cycles needed. Third, a task’s execution time becomes $$T_{vm,i} \left( t \right) = {\raise0.7ex\hbox{${s_{i} }$} \!\mathord{\left/ {\vphantom {{s_{i} } {C_{k} \left( t \right)}}}\right.\kern-0pt} \!\lower0.7ex\hbox{${C_{k} \left( t \right)}$}}$$ when it is scheduled on a particular $$VM k$$. where $$VM k$$ computational rate is denoted by $$C_{k} \left( t \right)$$.

Finally, the energy consumption is divided into three categories: local energy, $E_{local,i} = e_{act} T_{local,i}$; transmission energy, $E_{tr,i} = e_{tran} T_{tr,i}$; and VM holding energy, $E_{vm,i} = e_{idle,i} T_{h,i}$, where the coefficients $e_{act}$, $e_{tran}$, and $e_{idle}$ denote the device activity, transmission, and idle rates, respectively.

Together, these presumptions create a tractable system model that connects energy use, computation, and transmission. The study can clearly capture the trade-offs of running tasks locally, offloading them to edge virtual machines, or sending them to the cloud by formally defining time and energy in this organized manner.

### Decision variables and notation

The decision-making framework of the system is defined through a set of binary and categorical variables. The primary decision variable is $${\text{K}}_{i} \left( {\text{t}} \right) \in \left\{ {0,1} \right\}$$, which indicates whether task $$i$$ at time $$t$$ is executed locally 0 or offloaded 1. When offloading occurs, an additional assignment variable $$x_{ik}$$$$\left(t\right)\in \left\{0,1\right\}$$ specifies whether task $$i$$ is allocated to $$VM k$$, with the restriction that at most one VM can be selected. The admissible set of feasible VMs for task $$i$$ is denoted as $$A\left(t\right)$$, reflecting resource constraints such as CPU, memory, and storage. Queue states are also incorporated into the notation, with waiting time defined as $$W_{i} = { }T_{a,i} {-}T_{c,i} { }$$where $$T_{a,i}$$ is the arrival time and $$T_{c,i}$$ is the completion time, enabling the integration of delay indicators into the performance model.

These choice variables act as the optimization problem’s control levers, directly influencing whether tasks are forwarded, offloaded, or executed locally. The model accurately captures the intricacy of resource allocation in MEC by fusing queue-based timing parameters, assignment limitations, and binary offloading indicators.

### Problem formulation

This section demonstrates the process of dispatching and executing tasks at the edge layer. The definitions of significant symbols utilized in relationships are provided in Table 2 for clarity. Assuming, time is divided into time slices t, which are in the form $\text{t}\in \{1,...,\text{n}\}$, the initial set of tasks $\text{M}\in \{\text{0,1},\dots ,\text{N}\}$ and where M = 0, that means there is no task in the set.

**Table 2:** Symbols and notations.

Symbol	Description
$d\left(t\right)$	Data size processing programs (eg and data)
$S\left(t\right)$	The size of processes (for example, the number of processor cycles)
${c}_{n}^{l}$	CPU capacity in local execution
${c}_{n}^{m}$	Power system running on the edge
${c}_{idle}$	The energy consumed by the mobile phone
${e}_{tran}$	Energy used for transmission
${e}_{active}$	The available energy of the mobile device
${c}_{vm}\left(t\right)$	Ability to calculate by the nth virtual machine processor
${h}_{n}$	State of decision to load
$H$	Set of tasks to load
${I}_{n}$	A set of executed tasks
${u}_{n}^{l}$	The ability to perform a task locally
${u}_{n}^{m}$	Ability to execute a task at the edge
$m$	Number of virtual machines
$n$	The number of tasks available in the $\text{i}-\text{th}$ virtual machine
$cap$	The capacity of a virtual machine
${t}_{s}$	Virtual gap
$\mu$	Service rate
$\lambda$	Queuing rate
$\phi$	Cost function
$\beta$	Weight parameter in the cost function
$W$	Knapsack capacity
$P$	The value of each task
${T}_{a}$	Start time to select a processor to run
${T}_{c}$	End time to execute a task on a processor

The collection $\text{H}\in \{{\text{h}}_{1}\}$ comprises tasks designated for execution on the mobile phone. We denote ma $\text{k}\left(\text{t}\right)\in \left\{\text{0,1}\right\}$ as the decision parameter. If $\text{k}(\text{t})=0$, jobs are dispatched to the edge; if $\text{k}(\text{t}) =1$, tasks are done locally on the mobile device $X$. The ideal response set is depicted, with ew[i] denoting the weight of the i-th job. In our method, energy consumption is a constant value determined by the weight of the GASO sums of their jobs and the associated profit of those activities. Chooses the interface with the greatest weight for loading onto layer 2.

The cumulative weight of the jobs does not exceed the Knapsack’s capacity, denoted as W, and the variable $0\le {\text{x}}_{\text{i}}\le 1$ is defined such that ${x}_{i}\times ew\left[i\right]$ represents the weight occupied by each task in the Knapsack^38^, whereas ${\text{x}}_{\text{i}}\times {\text{p}}_{\text{i}}$ signifies the corresponding value^39^. Each problem illustrates the objectives of the GASO algorithm as presented in (1), (2) and (3).

1 $${\text{MAX}}\mathop \sum \limits_{{{\text{i}} = 1}}^{{\text{n}}} ({\text{x}}_{{\text{i}}} \times {\text{p}}_{{\text{i}}} )$$

2 $$\mathop \sum \limits_{{{\text{i}} = 1}}^{{\text{n}}} {\text{x}}_{{\text{i}}} \times {\text{ew}}\left[ {\text{i}} \right] \le {\text{W}}$$

3 $${\text{p}}_{{{\text{i}} = }} \frac{{{\text{e}}\left[ {\text{i}} \right]}}{{{\text{ew}}\left[ {\text{i}} \right]}}$$

In (3), ${\text{p}} \in \left[ {1,2, \ldots ,{\text{n}}} \right]$ denotes the value of the i-th job, where ${\text{p}} \in \left[ {1,2, \ldots ,{\text{n}}} \right]{ }$ represents the set of values for all tasks chosen for loading, and $e\left[ i \right]$ signifies the utility of the *i-th* work.

### Dynamic scheduling

To enhance utility, scheduling is conducted according to a priority queue, with task queue priority derived from the output of the FPTS algorithm. We will examine the characteristics of energy consumption and execution time to enhance reliability in the effective edge environment. Our fuzzy system comprises three tiers: fuzzification tier, inference tier, and fuzzy destructibility tier. The input for the first level consists of tasks characterized by two parameters: energy usage and execution time. The output of the fuzzy level serves as the input for the inference level, and subsequently, the output of the inference level functions as the input for the third level. The input values are transformed into fuzzy data at the initial level. Fuzzy rules are established at the second level, and ultimately, the fuzzy destructibility level converts the fuzzy sets into linguistic form to yield a definitive output.

The system’s input is represented as $\text{M }(\text{ct}\times \text{et})$, comprising a collection of jobs characterized by two parameters: execution time and energy consumption. The system’s output is shown as an array $\text{p}[\text{1,2},\dots ,\text{n}]$, corresponding to ct and et, respectively. ${\text{task}}_{\text{i}}^{\text{p}}$ signifies that this job possesses priority p. To convert the parameters into those suitable for inference in the fuzzy system, rules have been established at the inference level, comprising a collection of if else statements^40^. The input fuzzing is ascertained by the Gaussian membership function, as seen in (4).

4 $${\upmu }_{{\left( {{\text{A}}^{{\text{i}}} } \right)}} \left( {\text{x}} \right) = {\text{exp}}\frac{{({\text{C}}_{{\text{i}}} - {\text{x}})^{2} }}{{2{\upsigma }_{{\text{i}}}^{2} }}$$

In (4), the values of ${\text{c}}_{{\text{i}}}$ and ${\updelta }_{{\text{i}}}$ represent the centrality of the function, whereas x denotes a collection of two inputs: energy consumption and execution time, which pertains to i. Amin represents the fuzzy set ${A}^{i}$, with the membership function output defined as ${\mu }_{{A}^{i}}\left(x\right)=\left[\text{0,1}\right]$. Gaussian fuzzy membership functions are prevalent in fuzzy logic literature, as they facilitate communication between fuzzy systems and neural networks. are employed utilizing Radial Basis Function (RBF). The regulations employed in our system are presented in Table 3.

**Table 3:** Fuzzy rules based on execution time and energy consumption.

	Energy consumption				
Execution time	very high	Up	Average	Down	very low
very high	very high	Up	Average	Average	Down
Up	Up	Up	Average	Average	Down
Average	Up	Average	Average	Down	very low
Down	Up	Average	Down	very low	very low
very low	Average	Down	Down	very low	very low

The output of the priority determination system is derived from the inputs of execution time and energy consumption, serving as the output of the fuzzy system[41]. The scale elements are contained in the set A {very high, high, medium, low, very low}, and the output is generated by multiplying the fuzzy sets. We presume the existence of two input sets, A and B. The multiplication of fuzzy sets is represented by $\text{A}\cap \text{B}$ which is ${\upmu }_{\text{A}}\left(\text{X}\right)\cap {\upmu }_{\text{B}}\left(\text{X}\right)=\text{min}({\upmu }_{\text{A}}\left(\text{X}\right)).$ The virtual machine replicates the data and apps of a smart device upon receiving a user request, while the control program identifies the most suitable machine for servicing. It is intended for loading. $d\left(t\right)$ denotes the magnitude of the program’s input data, while $S\left(t\right)$ represents the extent of the computations^42^. Both vectors are independently and identically dispersed in each time slot t. The execution time at the boundary is determined using Eqs. (5) and (6).

5 $${\text{T}}_{{{\text{MEC}}}} \left( {\text{t}} \right) = {\text{T}}_{{\text{r}}} + {\text{T}}_{{\text{h}}} + {\text{T}}_{{{\text{tr}}}}$$

6 $${\text{T}}\left( {\text{t}} \right) = \mathop \sum \limits_{{{\text{n}} \in \vartheta }} {\text{k}}\left( {\text{t}} \right){\text{T}}_{{{\text{MEC}}}} + [1 + \mathop \sum \limits_{{{\text{n}} \in \vartheta }} {\text{K}}\left( {\text{t}} \right){\text{T}}_{{{\text{local}}}} ]$$

Here, ${\upmu }_{{\text{A}}} \left( {\text{X}} \right) \cap {\upmu }_{{\text{B}}} \left( {\text{X}} \right) = {\text{min}}\left( {{\upmu }_{{\text{A}}} \left( {\text{X}} \right).{\upmu }_{{\text{B}}} \left( {\text{X}} \right)} \right)$ represents the duration required to verify a request in the nth virtual machine, whereas ${\text{T}}_{{\text{r}}} \left( {\text{t}} \right)$ denotes the response time in addition to the request loading time from the virtual machine. Value ${\text{T}}_{{{\text{tr}}}} \left( {\text{t}} \right) = {\text{d}}\left( {\text{t}} \right)/{\text{c}}_{{\text{vm }}} \left( {\text{t}} \right),$ representing the transfer time of the task to be loaded into the virtual machine during time slot t, and ${\text{T}}_{{{\text{local}}}} \left( {\text{t}} \right) = {\text{d}}\left( {\text{t}} \right)/{\text{c}}_{{{\text{local}}}} \left( {\text{t}} \right)$ as indicated in (6). The task execution duration occurs locally on the device^43^.

We posit that $\text{k}(\text{t})\in \{\text{0,1}\}$ represents the chance of a job being done locally on the mobile device; otherwise, the task is executed via offloading to the edge. To compute the energy consumption, we define ${\text{E}}_{\text{MEC}}\left(\text{t}\right)={\text{E}}_{\text{tr}}\left(\text{t}\right)+{\text{E}}_{\text{vm}}(\text{t})$. This illustrates the energy expended during task transfer and execution within the virtual machine^43^. In this context, ${\text{E}}_{\text{tr }}= {\text{T}}_{\text{tr}}\times {\text{e}}_{\text{tran}}$ and ${\text{E}}_{\text{vm}}={\text{T}}_{\text{h}}\times {\text{e}}_{\text{idle}}$, the total energy consumption is obtained from Eq. (7).

7 $${\text{E}}\left( {\text{t}} \right) = \mathop \sum \limits_{{{\text{n}} \in \vartheta }} {\text{K}}\left( {\text{t}} \right){\text{E}}_{{{\text{MEC}}}} + \left[ {1 + \mathop \sum \limits_{{{\text{n}} \in \vartheta }} {\text{K}}\left( {\text{t}} \right){\text{E}}_{{{\text{local}}}} } \right]$$

In (7), ${\text{E}}_{\text{local}}={\text{T}}_{\text{local}}\times {\text{e}}_{\text{active}}$ represents the energy consumption in the smart workplace. The system’s overall energy usage throughout each time period is shown in Eq. (7). The energy required when tasks are moved to and carried out at the MEC layer is taken into consideration in the first section, and the energy spent when tasks are carried out locally on the device is covered in the second. The equation accounts for the total cost of both execution modes since the decision variable $\text{K}\left(\text{t}\right)$ indicates whether a job is run locally or offloaded.

The outcomes of these values are delineated as assessment parameters in sect. “Evaluation”. The objective is to achieve optimal performance with the minimal number of processors. The waiting time refers to the average duration a task remains in the line, whereas the likelihood of delay corresponds to the ratio of tasks entering the waiting queue. We establish a threshold value for the service level. The minimal number of processors needed is the quantity necessary to achieve a service level that meets or exceeds the threshold target^45^. When tasks reach the edge, they undergo a waiting period before execution, which is determined by Eq. (8).

8 $${\text{W}}_{{\text{C}}} = {\text{T}}_{{\text{C}}} - {\text{T}}_{{\text{a}}}$$

In (8), $T_{C}$ denotes the arrival time of a task, while ${T}_{a}$ signifies the time of processor allocation for execution. The quantity of received requests reflects the number of requests dispatched from each task, which may signify the volume of network traffic. A diminished request rate signifies reduced network traffic, whereas an elevated request rate indicates increased network traffic. When the network traffic $\lambda$ is proportionate to the login requests^40^, the system achieves equilibrium, and the system balance factor is derived from Eq. (9).

9 $${\text{L}} = \frac{{{\text{n}}_{{\text{i}}} }}{{{\text{cap}}_{{\text{i}}} }}$$

In (9), $n_{i}$ denotes the quantity of jobs in the $ith$ virtual machine, whereas $cap_{i}$ represents the capacity of that virtual machine^41^. When the system is unbalanced, the system load escalates. The system load is derived from Eq. (10).

10 $$\sqrt {\mathop \sum \limits_{{{\text{i}} = 1}}^{{\text{m}}} \frac{{({\text{L}}_{{\text{i}}} - {\text{L}}_{{{\text{avg}}}} )^{2} }}{{\text{m}}}}$$

where m represents the quantity of virtual machines and $L_{avg}$ is the average load on each virtual machine. To compute the cost function, we assign a value to the weight parameter. The parameter β is defined as a weight parameter within the interval [0,1]. If $\beta = 1,$ energy consumption does not influence the decision to load to the edge^42^. The cost function for energy consumption and execution time is obtained according to Eq. (11)

11 $$\Phi \left( {\text{t}} \right) = {\upbeta }\frac{{{\text{T}}\left( {\text{t}} \right)}}{{{\text{T}}_{{{\text{local}}}} \left( {\text{t}} \right)}} + {\upbeta }\frac{{{\text{E}}\left( {\text{t}} \right)}}{{{\text{E}}_{{{\text{local}}}} \left( {\text{t}} \right)}}$$

##  Proposed method

This section presents the ideal loading and scheduling algorithm for both the device and the edge. Resource allocation is also conducted on the periphery. Consequently, our proposed model comprises three algorithms implemented in three stages:

- GASO Optimized device side loading algorithm
- FPTS dynamic scheduling algorithm on the edge side
- Algorithm of allocation of resources on the edge side

**Fig. 2:**
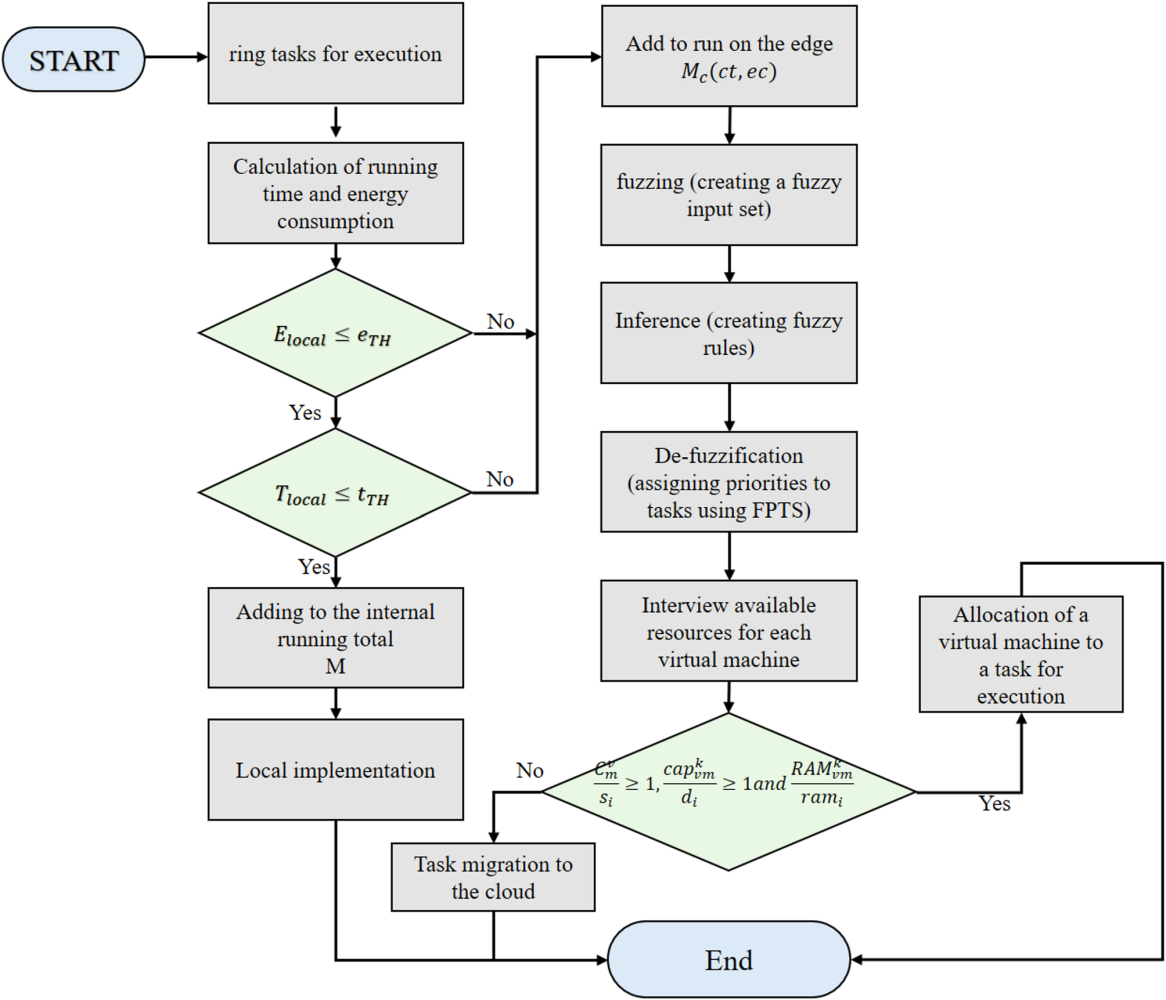
Task division flows chart.

The specifics of the algorithms are elaborated in the subsequent subsections. Figure 2 illustrates the execution of work and the allocation of tasks within a straightforward workflow.

Figure 2 presents a detailed flowchart that outlines the task division process within the proposed MEC framework, summarizing the operational workflow for effective task offloading and scheduling. The flowchart delineates the decision-making process, beginning with task initiation on the mobile device, subsequently employing the GASO algorithm to categorize tasks according to energy consumption and execution time parameters. Tasks are assessed for their suitability for local execution or offloading to the edge layer, where the Fuzzy-based Priority Task Scheduling (FPTS) algorithm employs fuzzy logic to prioritize them for optimal resource allocation.

### GASO edge loading algorithm

The details of the GASO pseudocode steps are shown in Algorithm 1. We possess a collection of n tasks from the set $\left\{1000,n\right\}$. Initially, we shall arrange the activities according to their value and profit, ensuring that $p[1]\ge p[2]\ge \dots \ge p[n]$, and compute the value of $\text{p}\left[1\right]\ge \text{p}\left[2\right]\ge \dots \ge \text{p}[\text{n}]$ based on the connection $e[i]/ew[i]$. Indeed, according to the weight, the inequality $p[i+1]/e[i+1] \le p[i]/e[i]$ holds true. Tasks with a larger percentage of energy usage are prioritized for loading. In each iteration, a task is chosen from the available tasks; if the value of $ew\left[i\right]$ is less than or equal to W, then $x\left[i\right]$ is set to 1 and W is reduced by $ew\left[i\right]$. The set $X$ comprises the response array containing the jobs with the highest values. Given that the jobs were organized initially, a for loop of $O\left(n\right)$ difficulty is conducted, resulting in an overall algorithmic complexity of $O\left(n\text{log}n\right)$ when combined with previous phases.

**Algorithm 1:**
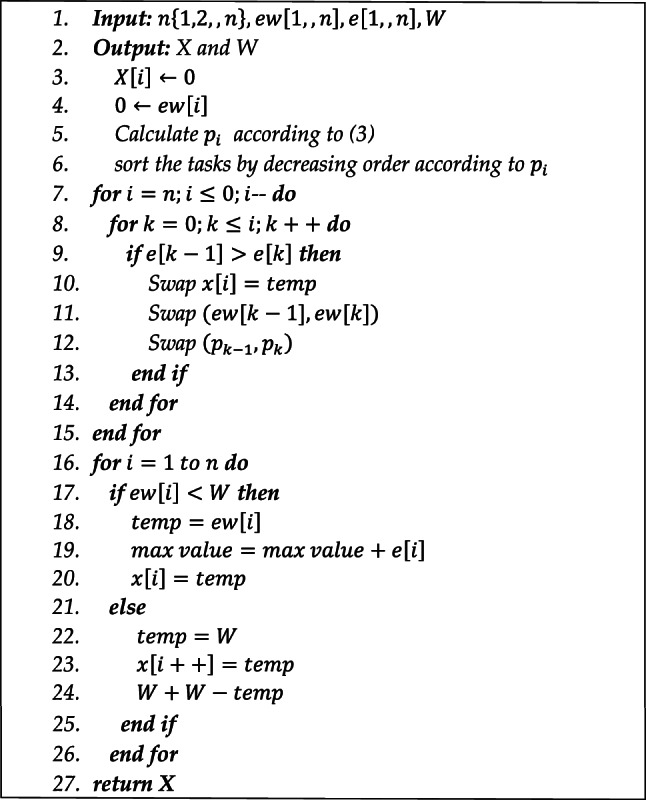
GASO loading method

### FPTS timing algorithm

This section describes the procedures of the FPTS algorithm. Algorithm 2 shows the procedures of the FPTS. We create a framework of rules and subsequently rank jobs with fuzzy logic. For every task involving energy consumption and execution duration. Extremely high priority is assigned to tasks with minimal energy consumption and execution time, while those with very low priority exhibit significantly reduced energy usage and execution duration. The task priority is determined by multiplying the priority levels of energy consumption and execution time; for instance, very low multiplied by very high results in a diminished priority.

Tasks characterized by minimal energy use and prolonged execution time are assigned low priority. To assign resources, each task request comprises three parameters: memory, processor, and data space, which are compared against the available values. Three sets of virtual machines ${\text{V}}_{1}\{\text{1,2},\dots ,\text{n}\}$, ${\text{V}}_{2 }\{\text{1,2},\dots ,\text{n}\}$, and ${\text{V}}_{3}\{\text{1,2},\dots ,\text{n}\}$ each comprise virtual computers equipped with adequate memory, processing power, and data capacity. The selection of an accessible vehicle is governed by the subsequent two policies:

- All virtual machines are busy and ${\text{V}}_{1} \cap {\text{V}}_{{2{ }}} \cap {\text{V}}_{3} \ne \Phi$, in which case the task migrates to the cloud.
- There is more than one active virtual machine and ${\text{V}}_{1} \cap {\text{V}}_{{2{ }}} \cap {\text{V}}_{3} = \Phi$, which is based on these three allocation parameters.

The input of this method is the sum of $X$, derived from the output of the GASO algorithm. All tasks are inputted into the FPTS interface, where they are transformed into fuzzy inputs based on execution duration and energy expenditure. Upon ranking the jobs, resources are allocated such that each task receives a minimum of one resource, or more, in relation to the available resources. This indicates that the following relationships must be established: ${\text{cap}}_{\text{vm}}^{\text{k}}/{\text{s}}_{\text{i}}\ge 1$, ${\text{cap}}_{\text{vm}}^{\text{k}}/{\text{d}}_{\text{i}}\ge 1$, and ${RAM}_{\text{vm}}^{\text{k}}/{\text{ram}}_{\text{i}}\ge 1$. Any virtual machine that meets one of these conditions is selected, and ultimately the pool of these machines is selected as a suitable resource for allocation. If there are no active resources, the tasks are sent to the cloud.

**Algorithm 2:**
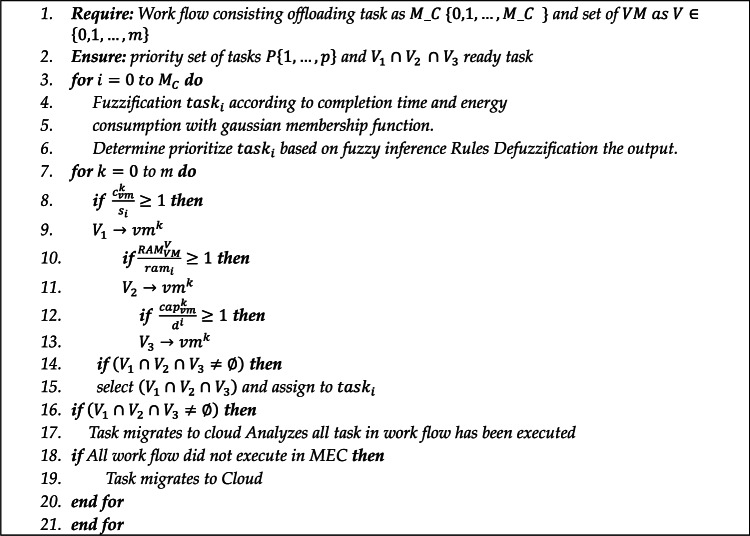
FPTS scheduling method

## Evaluation

This section reviews and analyzes the system model, presentation, and numerical results of the employed algorithms, with the findings organized into two subsections: assessment environment and analysis. In this section, the details of the system model, presentation, and numerical results of the algorithms used are reviewed and analyzed, and the results are presented in two sections: evaluation environment and analysis.

### Evaluation environment

This section evaluates the loading, scheduling, and resource allocation strategies of the proposed system. We assess the efficacy of the suggested model utilizing a 10-core Intel R processor operating at a frequency of 6.3 GHz and equipped with one GB of RAM. The Windows 10 operating system is 64-bit. We utilize two virtual machines as edge-to-edge nodes and a smartphone device. Each machine is equipped with 2 MB of memory and a 5-core processor. The total hard disk capacity is 1 TB, with each virtual machine utilizing 200 GB of space.

The Samsung F720 smart device model features an 8-core processor operating at 2 GHz, 2815 MB of RAM, and supports 4G and Wi-Fi connectivity. The smartphone operates on Android 8 and has a battery capacity of 3600 mAh, initially at 92% charge.

The power of the passive, active, and transmission batteries is 79%, 1.5%, and 2.2%, respectively. We establish a link between the edge and the device via the Wi-Fi network. Two categories of applications operate on the smart device. An application operates GASO. The tasks derive from the augmented reality and facial recognition algorithms, each possessing two metrics: energy consumption and execution time. The energy consumption of Android functions is quantified within the program’s Android code.

The application on the mobile device use GASO’s offloading algorithm to classify processes for either local execution or offloading. The tasks are thereafter dispatched to the virtual machines designated as the edge level inside our architecture, whereupon their arrival, they are prioritized by a program employing the FPTS scheduling algorithm, arranged in descending order of priority. are carried out The device and virtual machine monitor evaluation parameters including waiting time, delay, and service rate, which are subsequently analyzed using R Studio software with C programming language. The imprecise part of this system has also been implemented in MATLAB software. The research results have also been analyzed using R software.

Furthermore, we derived the assessment parameters for a scheduling and prioritization job from prior research and utilized the graph table in this software to juxtapose our results. The graph clearly illustrates the upward and decreasing trends of our research results through numerical comparison. In many instances, we employed a numerical table to present the specifics of the data acquired. The last section of the work, which demonstrates the utilization of the mobile device’s resources, was executed by invoking Android functions within the application code during task loading. In the context of the cost function, we establish the value of $\upbeta =0.5$. The GASO algorithm is configured with capacity values of 200 and 500.

**Fig. 3:**
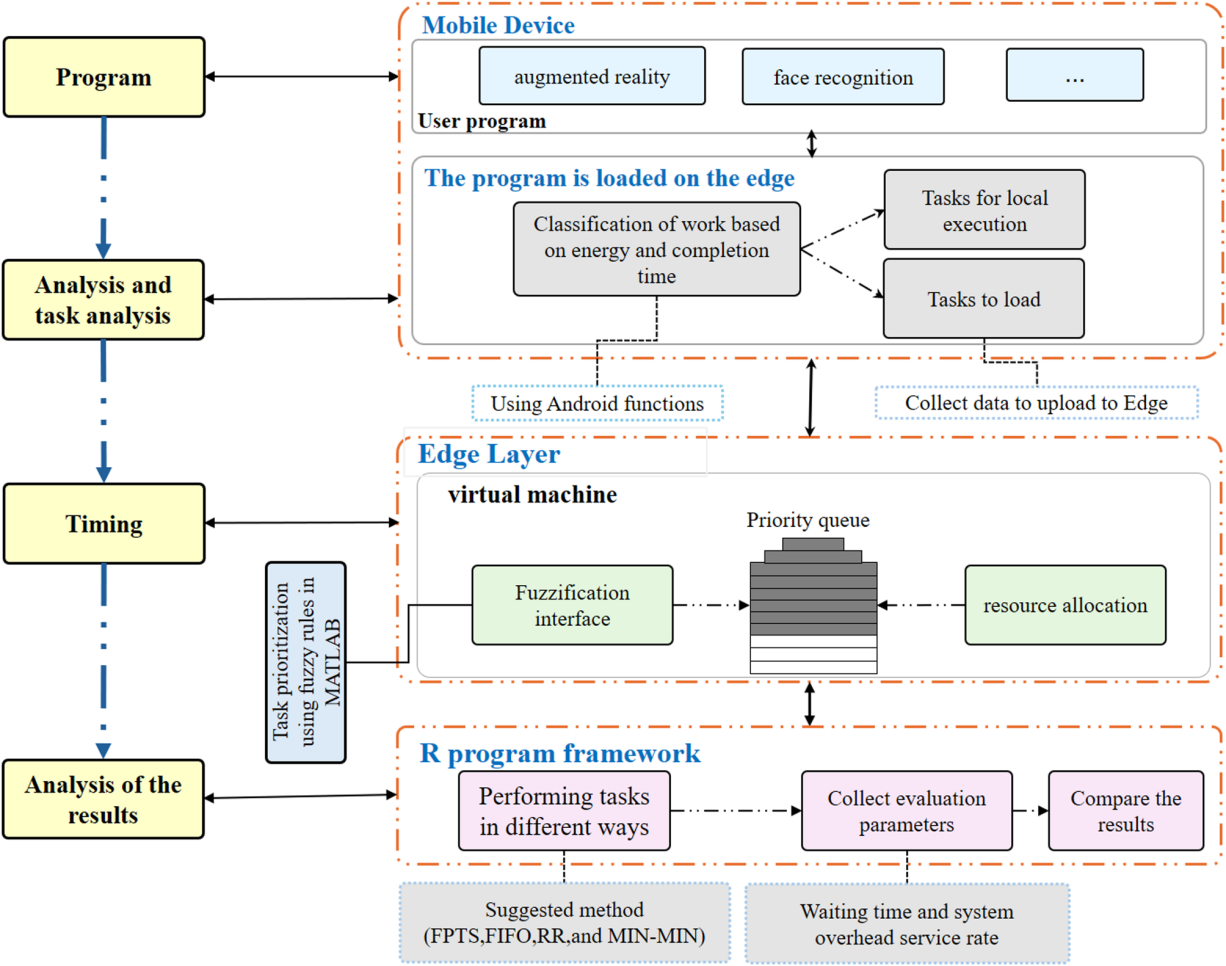
The system and structure of the proposed work steps.

Figure 3 presents a comprehensive schematic representation of the system architecture and workflow procedures for the proposed MEC framework, illustrating the organized integration of task offloading, scheduling, and resource allocation operations. The illustration represents a three-tier architecture comprising mobile devices, edge computing nodes, and conventional cloud infrastructure. The diagram illustrates the sequential workflow, commencing with task generation on mobile devices, wherein the GASO algorithm assesses workloads according to energy consumption and execution time to facilitate optimal offloading decisions. Subsequently, jobs are delegated to the edge layer, where the FPTS algorithm employs fuzzy logic to prioritize and assign work to virtual machines, therefore improving resource utilization and reducing latency. This flowchart depicts the conditional migration of tasks to the cloud when edge resources are inadequate, hence guaranteeing system equilibrium and efficiency.

#### Dataset and parameter settings

Two genuine application kernels that are frequently used in MEC studies augmented reality (AR) and facial recognition (FR) were utilized to create the experimental workloads. Both were deployed on Android smartphones, and the system profiler was used to assess energy consumption. With mean arrival rates $\lambda \in \left\{\text{10,20,40}\right\}tasks/s$, task arrivals follow a Poisson process. The execution time (in CPU cycles) and energy requirement (in Joules) of each task are specified. To assess generality, we also created a synthetic mixed dataset that incorporates tasks with different sizes and energy intensities.

The following algorithmic settings were set: capacity budget $W\in \left\{\text{300,600}\right\}$, number of processors $\in \left[\text{1,8}\right]$, and weight factor $\beta \in \left\{\text{0.3,0.5,0.7}\right\}$. The baseline optimizers^24,30^, and^28^ were set up using the hyperparameters that were suggested. Every experiment was conducted 20 times, and the results are shown as mean ± standard deviation. The datasets used in the tests, which include a synthetic mixed workload and AR and FR application kernels, are shown in Table 4 Every job adheres to a Poisson arrival process (λ = 10–40), guaranteeing accurate modeling of MEC environments with varying energy requirements and execution times.

**Table 4:** Summary of workload datasets used in the evaluation, including task counts, average execution time, energy demand, and arrival process.

Dataset	No. of tasks	Avg. execution time (CPU cycles)	Avg. energy demand (J)	Arrival process	Notes
Augmented reality (AR)	10,000	$1.2 \times 10^{6}$	0.85	Poisson $\left( {\lambda = 10{-}40} \right)$	Mobile AR rendering kernels implemented on Android
Face recognition (FR)	10,000	$1.8 \times 10^{6}$	1.10	Poisson $\left( {\lambda = 10{-}40} \right)$	CNN-based face recognition kernels on Android
Mixed synthetic workload	12,000	$1.5 \times 10^{6}$	0.95	Poisson $\left( {\lambda = 10{-}40} \right)$	Combination of light, medium, and heavy tasks

### Analysis of the results

**Fig. 4:**
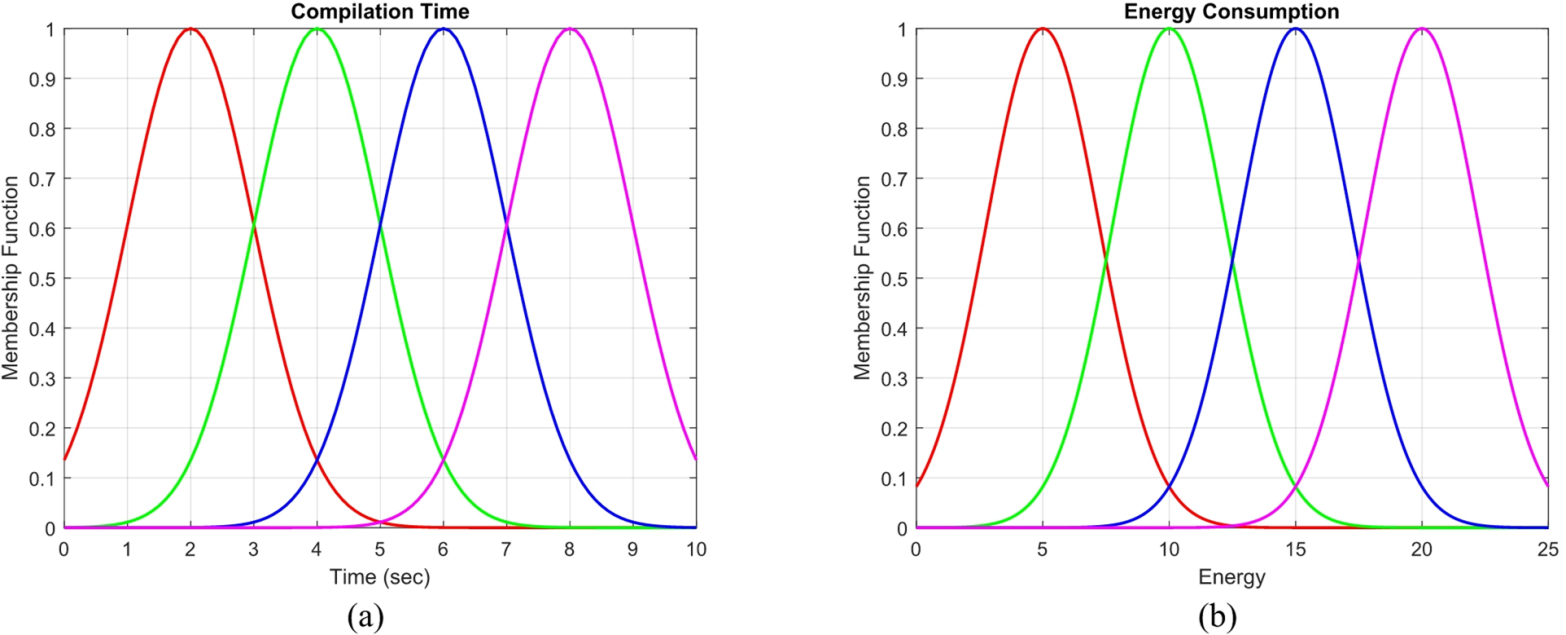
Fuzzy membership function of input sets.

**Fig. 5:**
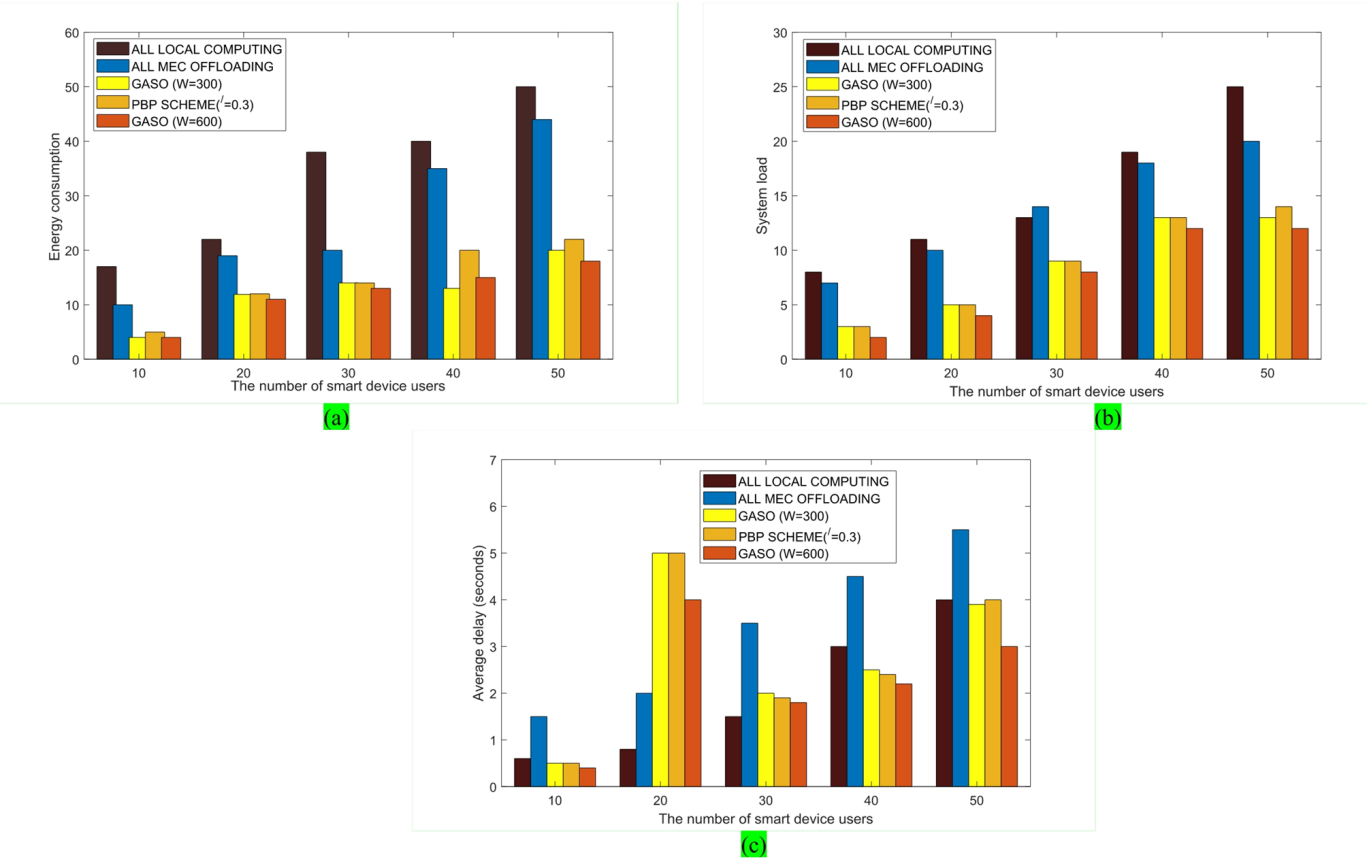
Comparison of various parameters across different user counts in five modes of the proposed method, with capacities of 600 and 300, PBP method, local execution, and complete task uploading to the edge: (**a**) the impact of user count on energy consumption, (**b**) the influence of user count on system load, (**c**) the effect of user count on delay.

This section begins with the evaluation of the loading problem based on the pseudocode shown in Algorithm 1. The state of the proposed GASO algorithm is juxtaposed with various additional scenarios. We also apply our approach in a scenario where tasks are randomly selected and unordered. Subsequently, we investigate the scheduling algorithm on the edge side according to the pseudocode shown in Algorithm 2 and the proposed model with different parameters. The benchmark FPTS algorithm is compared with three algorithms: RR, FCFS, and SJF. In FIFO, tasks are processed in the order they enter the queue. In RR, each activity is performed within a specified time interval. SJF is a scheduling algorithm that prioritizes tasks based on the shortest execution time for subsequent processing. Figure 4 shows the membership functions of the FPTS algorithm based on the input parameters of execution time and energy consumption. Ultimately, we assess the resource allocation aspect and ascertain the percentage of work migrated to the cloud. The proposed GASO algorithm is compared in two capacity cases of 200 and 500 with the mode of executing all tasks locally, offloading all tasks to the edge, and the PBP method where γ = 0.2. Figure 5a shows the energy consumption for these four modes and different numbers of users. The energy consumption in GASO is lower than PBP for a large number of users. Also, the energy consumption for offloading all tasks to the edge is lower than for executing all tasks locally.

The energy usage associated with offloading all tasks to the edge is less than executing all tasks locally. The proposed approach, with a capacity of 600, demonstrates optimal value when the user count exceeds 20. Utilizing GASO is more efficient than delegating all duties to the edge and is more effective for a substantial user base. Figure 5b illustrates that, in the PBP scheme outlined in this study, the system load escalates with an increase in the number of users. GASO offers superior system overhead performance compared to alternative modes, representing the highest system overhead for local execution. Figure 5c illustrates that the delay in local implementation is inferior to that of all other options.

**Fig. 6:**
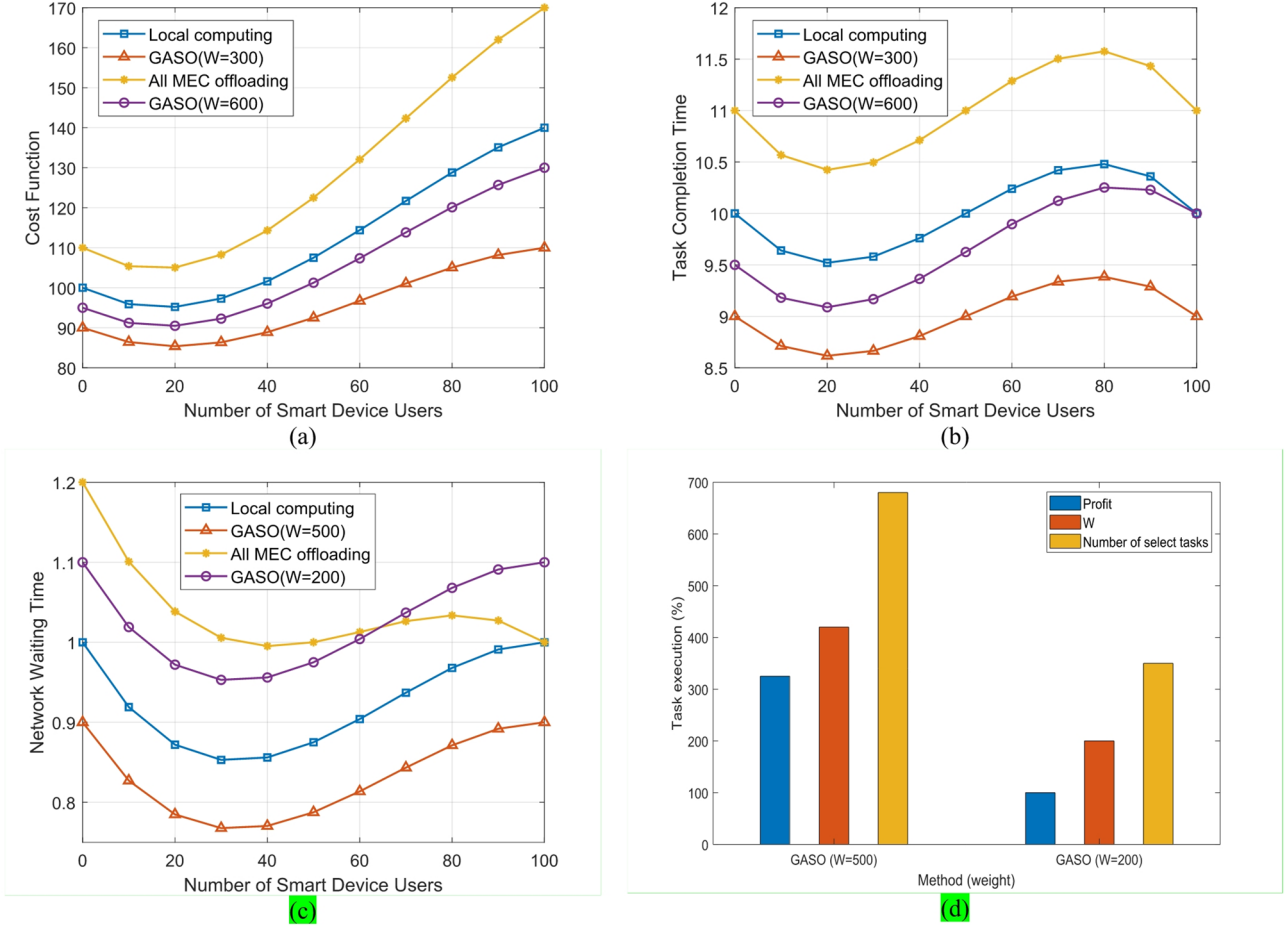
Comparison of various parameters for differing numbers of phone users across four schemes: GASO(W = 300), GASO(W = 600), local execution, and offloading all tasks to the edge. This includes (**a**) the impact of user quantity on the cost function, (**b**) the influence of user quantity on task completion time, (**c**) the effect of user quantity on network waiting time, and (**d**) specifics of the GASO algorithm.

Figure 6a illustrates the performance comparison of the suggested method with capacities of 300 and 600local executions, with the uploading of all jobs to the edge. For (W = 300) GASO, the cost function is minimized, being slightly lower than the cost function associated with executing all activities locally and offloading all duties to the edge. As W in GASO rises to 600, the cost function diminishes due to the capacity to allocate additional tasks to the edge. Execution and completion duration of a comprehensive task, encompassing specifics such as execution time on a mobile device, local execution wait time, transfer wait time, transfer duration, edge execution wait time, and execution time.

The waiting time for transfer in local implementation is zero due to the absence of transfers, however the proposed loading technique exhibits an ideal value as transfer demand increases. When W reaches 600, the GASO algorithm is optimal for the execution time parameter. Indeed, the waiting time on the device side is diminished in comparison to local execution for a substantial number of users. Figure 6b illustrates a comparison of this case. Figure 6c shows that the peak of network utilization is when all tasks are transferred, followed by a scenario in our proposed algorithm.

The comparative outcomes of several scheduling techniques are reported in Table 5. The intelligent optimizers^24,28,30^, and the suggested GASO + FPTS are compared with the traditional methods (local execution, all-around execution on the edge, PBP). The findings demonstrate that GASO + FPTS consistently provides the lowest energy consumption, the lowest system load and latency, and the highest percentage of tasks executed at the edge, even while the intelligent baselines outperform the classical schedulers.

**Table 5:** Performance comparison of classical schedulers, intelligent baselines^24,28,30^, and the proposed GASO + FPTS in terms of energy, system load, delay, and edge execution rate.

Method	Energy consumption ↓	System load ↓	Avg. delay (s) ↓	Edge execution rate (%) ↑
Local execution	28.2	18.05	1.10	–
Run all on edge	21.3	16.24	0.96	100
PBP	12.5	10.7	0.95	85
GASO (W = 300)	10.1	10.5	0.90	89
GASO (W = 600)	9.2	9.8	0.82	92
PSO + GA[30]	11.0	11.5	0.88	88
NSGA-II[24]	10.5	10.2	0.85	90
DQN[28]	9.8	10.0	0.84	91

We utilize the network predominantly at 600. Figure 6d illustrates the specifics of the proposed algorithm in two modes. In GASO (W = 300), the filled weight is 188, 2/200, with 314 selected tasks, and the value is P = 117. In GASO (W = 600), the filled weight is 4.500435, accompanied by 668 selected jobs, and the value is p = 320.

**Fig. 7:**
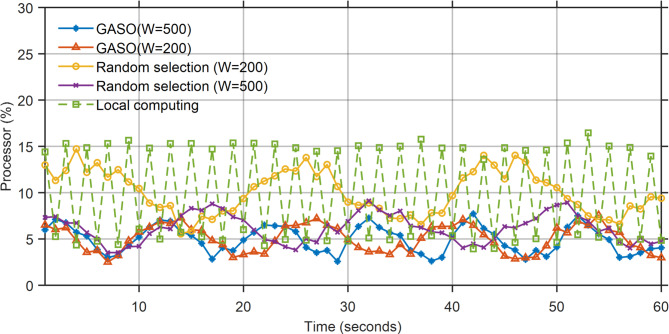
Processor status based on the performance of the proposed approach.

In GASO (W = 500), the proportion of filled versions is 435/500, with 668 jobs picked, and the value is p = 320. The information is presented in Table 6. In this part, we have the RAM and network processor task in several modes of the proposed method with capacity of 300 and 600, random selection of tasks (W = 600), random selection of GASO tasks (W = 300) and We measure the local execution of all tasks at time $1\le t\le 60$. In Fig. 7, the processor’s condition during the local execution of all processes is at maximum utilization capacity.

**Table 6:** GASO algorithm details.

Algorithm	Total value	Total weight	Number of tasks selected to load
GASO(W = 600)	320/9	435	668
GASO(W = 300)	117/6	189/2	314

**Fig. 8:**
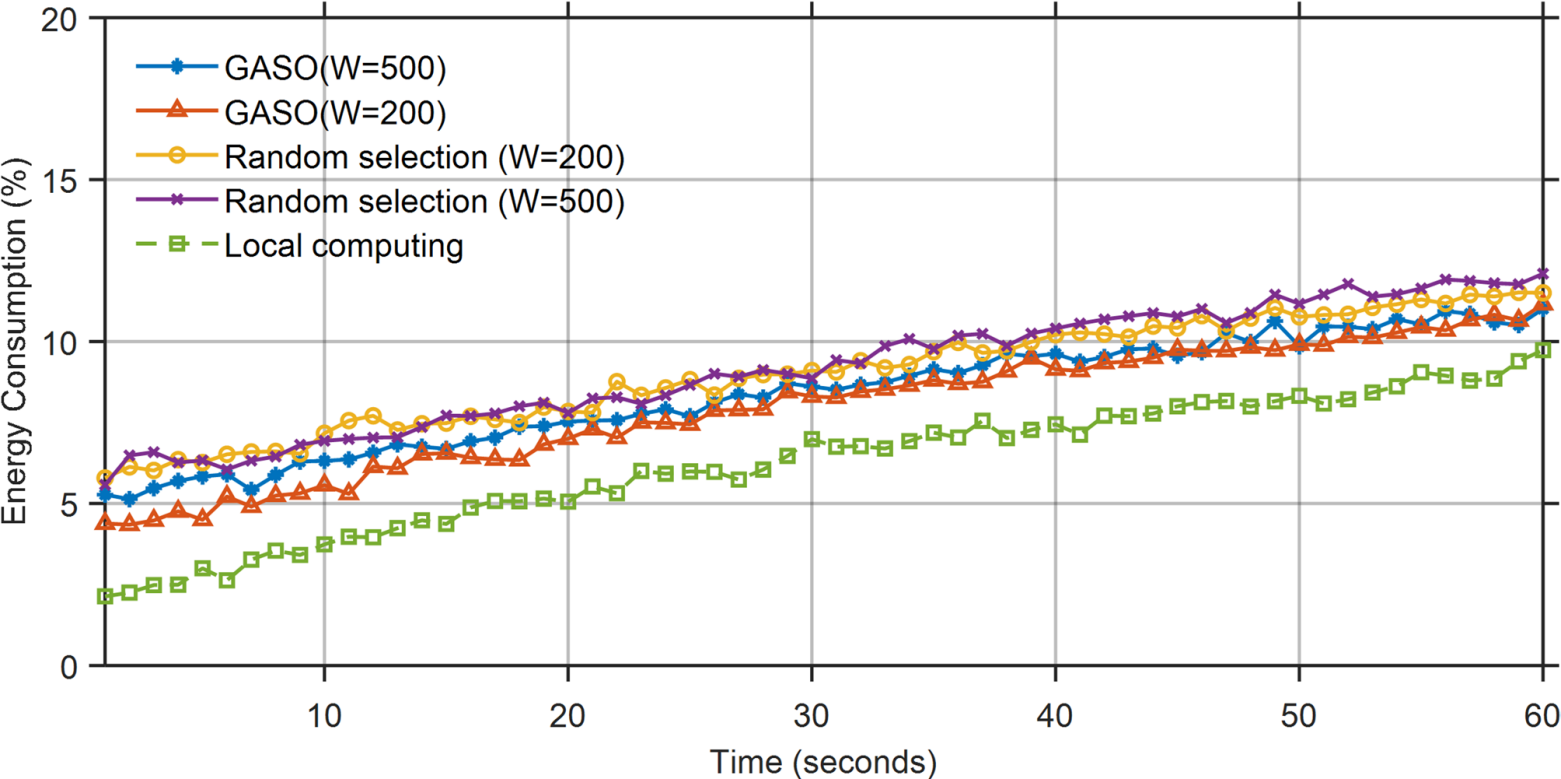
Energy consumption status based on the performance of the proposed approach.

**Fig. 9:**
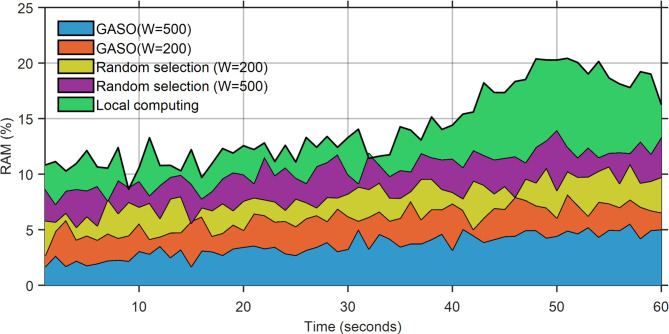
RAM status based on the performance of the proposed approach.

**Fig. 10:**
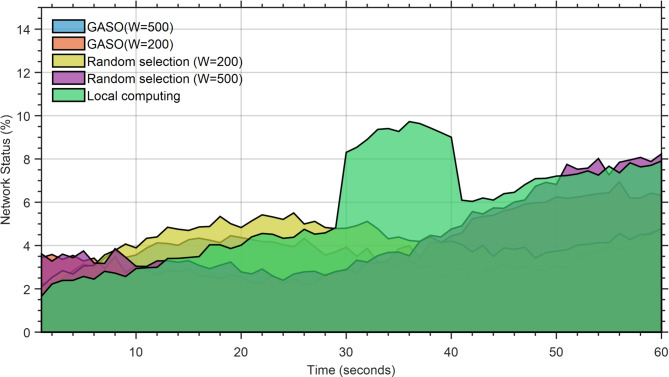
Network bandwidth status based on the performance of the proposed approach.

The battery usage during local execution exceeds that of the alternative mode, as all functions are performed on the device. The ideal battery consumption occurs in GASO mode (W = 600). The peak battery consumption in this mode is 7% at t = 60, equivalent to 19% in the randomly selected GASO mode (W = 600). Figure 8 illustrates the status of battery use. Figures 9 and 10 indicate that the RAM utilization during local execution is minimal, as no tasks are loaded, in contrast to the network usage scenario. Regarding the random selection of GASO(W = 300) and GASO(W = 600), The RAM usage status is optimal. Transmission status within the network during the specified time window, excluding times ${t}_{54}$ and ${t}_{54}$ for the four states.

The disparity has diminished significantly. The maximum network use for both modes occurs at W = 600, as an increased number of jobs are chosen for dispatch. Our objective is to minimize resource utilization, namely by achieving optimal efficiency with the fewest processors possible.

In FPTS, the maximum waiting time with a single processor is 5 s, however with three processors, the waiting time reduces to 0.5 s. For the FIFO and RR algorithms, the SJF values are 0.15 and 0.2, corresponding to 5 and 4.4 processors, respectively. Conversely, the FPTS algorithm achieves the optimal likelihood of minimal delay and maximal service quality with four processors. The likelihood of delay and service level with processor 8 exhibits an optimal value in FPTS, with minimal variation following the implementation of the suggested SJF algorithm.

However, for a significant number of jobs, it possesses an ideal solution. This algorithm minimizes waiting time, reduces the likelihood of delays and system overhead to an acceptable threshold, while simultaneously enhancing the service level considerably. This algorithm performs more effectively with a limited number of processors.

The suggested system utilizing FPTS achieves a stable state with the minimal number of processors in comparison to alternative techniques. The subsequent matter to be assessed pertains to resource allocation. The majority of tasks are performed at the edge; but, in instances of inadequate resources and delays, they are migrated to the cloud. The suggested approach and three benchmark methods were examined in this procedure, as seen in Fig. 10.

This flow was evaluated for data with arrival rates of 250 and 500. In the proposed algorithm, following SJF, a greater proportion of tasks are done on the edge, specifically 92.11% at a data input rate of 250 and 90.88% at a data entry rate of 500, respectively. The efficiency numbers for the SJF algorithm at the second level are 68.80% for an entry rate of 250 and 71.38% for an entry rate of 500.

**Fig. 11:**
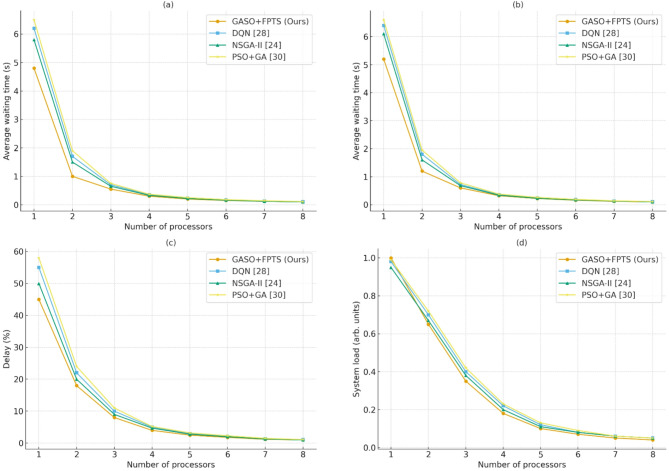
Comparison of evaluation parameters with the least number of processors for the proposed algorithm and benchmark algorithms, (**a**) average waiting time, (**b**) service level, (**c**) delay and (**d**) system load.

A thorough comparison of the suggested GASO + FPTS algorithm with intelligent and classical baselines along four performance dimensions is shown in Fig. 11. Panel (a) demonstrates the effectiveness of our approach in scheduling and prioritizing jobs under resource constraints by consistently achieving the lowest average waiting time, especially when the number of processors is limited. The service level is shown in Panel (b), where GASO + FPTS outperforms DQN^28^, NSGA-II^24^, and PSO + GA^30^ by maintaining above 93% completion even with fewer processors.

The suggested method maintains noticeably lower system load and delay percentages in panels (c) and (d) when compared to all baselines. Even if intelligent techniques like NSGA-II ^24^ and DQN^28^ reduce the performance gap in comparison to traditional schedulers, they still have higher overhead and variation. In contrast, GASO + FPTS maximizes task execution at the edge while minimizing system strain through consistent and balanced resource consumption.

**Fig. 12:**
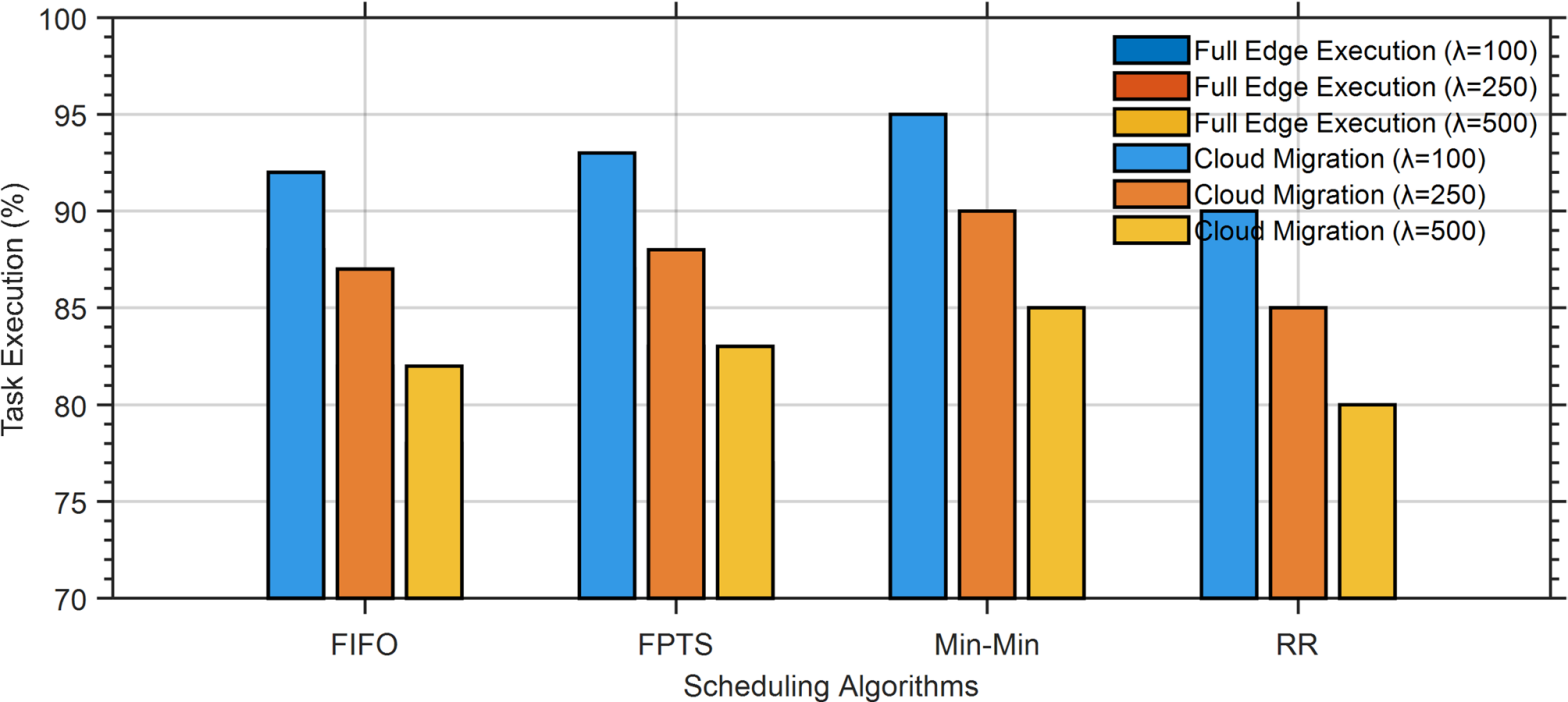
Execution of tasks at the edge and migration to the cloud based on the performance of the proposed approach.

Task execution rates at the edge and during cloud migration under various scheduling techniques are shown in Fig. 12. The findings demonstrate that, especially when subjected to high loads (λ = 500), edge-based execution consistently outperforms cloud migration in terms of job completion rates. When it comes to maintaining high execution percentages, the suggested FPTS outperforms FIFO and RR and performs on par with or better than Min-Min.

Table 7 presnts comprehensive performance measurements for the suggested FPTS, contemporary intelligent optimizers^24,28,30^, and conventional schedulers (FIFO, SJF, RR). The findings verify that although PSO + GA^30^, NSGA-II^24^, and DQN^28^ significantly surpass traditional techniques, the suggested FPTS continuously attains the lowest system load and delay, along with the highest service level and the least amount of waiting time, especially when processor availability is constrained.

**Table 7:** Comparison of scheduling algorithms in terms of system load, delay, service level, and waiting time across different processor counts.

Algorithm	System load	Delay (%)	Service (%)	Waiting time (s)	#Processors
FPTS (ours)	16 → 0.002	93 → 0.35	82.08 → 99.9	8 → 0.005	1 → 6
FIFO	860.36 → 0.002	98 → 2.36	75.21 → 99.97	3.9 → 0.005	3 → 9
SJF	7.618 → 0.001	99.9 → 1.4	79 → 99	1 → 0.003	1 → 7
RR	42.25 → 0.001	93 → 2.1	65 → 99	1 → 0.001	2 → 8
PSO + GA^30^	12.0 → 0.005	92 → 1.8	80 → 98.5	0.15 → 0.02	2 → 5
NSGA-II^24^	10.0 → 0.004	91 → 1.5	82 → 99	0.12 → 0.015	2 → 5
DQN^28^	9.5 → 0.003	90 → 1.2	83 → 99.2	0.10 → 0.012	2 → 5

### Complexity and correctness analysis

The suggested GASO offloading algorithm picks jobs in $O\left(n\right)$ and organizes them by utility ratio in $O(n log n)$ time, resulting in an overall complexity of $O\left(n\text{log}n\right)$. Compared to metaheuristic optimizers that need iterative search, this is far faster. Greedy selection produces a near-optimal solution with polynomial complexity, which is crucial for real-time mobile edge situations, even though it does not ensure the exact global optimum for the 0–1 knapsack problem.

Fuzzification $O\left(n\right)$, inference $O\left(n\right)$, and allocation over mmm virtual machines $O\left(n\cdot m\right)$ are the three stages of the FPTS scheduler. As a result, its complexity increases with the number of tasks and processors and is polynomial. Since the method consistently yields a valid allocation, accuracy is guaranteed. Every task is either carried out locally, scheduled at the edge, or sent to the cloud in the event that resources are unavailable. Therefore, the approach shows near-optimal performance under the evaluated workloads and ensures workable solutions with finite cost, even though it sacrifices exact optimality.

## Conclusion

This article examines the loading and scheduling model on both the device and edge sides. The proposed model investigated the optimization of battery usage on the device side and the reduction of delay and waiting time at the edge service level. We introduced an optimal offloading policy that use the greedy knapsack method to identify the most advantageous group of jobs for offloading.

This technique categorizes tasks into two groups for local execution and edge loading. This technique aims to optimize the selection of jobs for edge loading, ensuring that the cost of executing activities on the edge remains comparable to local execution, hence facilitating resource conservation. GASO demonstrated the most favorable outcomes for reducing energy use.

The numerical findings indicated that the GASO algorithm enhanced energy usage by 28.2% relative to the local execution of all tasks, which exhibited a 21.35% improvement, and by 12.54% compared to the PBP approach. This algorithm demonstrates superior performance compared to the other assessed modes with an increase in user quantity. The FPTS algorithm offers an appropriate prioritizing of tasks based on execution time and energy consumption, enhancing task execution speed in edge environments relative to local device execution.

The execution of the prioritized job set in this schedule enhances waiting time, latency, and service level with a maximum of 5 processors, whereas these values are 8, 7, and 7 for FIFO, RR, and SJF algorithms, respectively. The system demonstrates that 92.11% of tasks are completed on the edge platform at a data input rate of 250, while 90.88% are executed at a data input rate of 500, with approximately 10% of jobs being sent to the cloud for execution. This study addresses a significant restriction of cloud computing, namely user distance and excessive traffic, by reallocating tasks to the intermediary layer at the edge.

Additionally, it eliminates the constraints of conventional scheduling techniques that prioritize jobs based solely on a single criterion, such as execution time, by employing phase-based dual-criteria prioritization that acknowledges the significance of both criteria. Delegating tasks to the edge aims to enhance execution speed relative to local processing and conserve device resources. Selecting the appropriate set of tasks, transferring them via established communication channels, and executing them within the edge environment constitutes a limitation of the job; failure to meet any of these criteria undermines the objectives of rapid and cost-effective implementation. The sources do not provide this information.

Given that tasks transitioned to the mobile computing environment encompass numerous criteria, including data volume and bandwidth usage, it is essential to evaluate these criteria and ascertain their significance for prioritization and scheduling issues. The matters are significant. In the future, it is essential to deploy the proposed method across numerous distributed virtual computers to prioritize the various jobs that arrive at the edge at any given instant using this way.

Investigating additional critical criteria for task prioritization is essential, as identifying these criteria and their interrelationships establishes their relative importance, ultimately facilitating task prioritization. This process enhances system efficiency and conserves mobile device resources. The topic of data security during transmission from the device to the edge and while operating on the edge platform warrants discussion.

## Data Availability

The datasets used and/or analyzed during the current study are available from the corresponding author upon reasonable request. In addition, all source codes implementing the proposed GASO offloading algorithm, the FPTS fuzzy-based priority scheduling, and the simulation pipeline have been openly released at GitHub and are permanently archived on Zenodo. 10.5281/zenodo.17077930
